# Impact of the reuse of reciprocating instruments on cyclic fatigue resistance: WaveOne Gold and R-Motion

**DOI:** 10.4317/jced.62925

**Published:** 2025-09-01

**Authors:** Gabriela Murat, Carlos Eduardo da Silveira Bueno, Carlos Henrique Meloni, Daniel Guimarães Pedro Rocha, Carolina Pessoa Stringheta, Alexandre Sigrist Martin, Rina Andrea Pelegrine, Wayne Martins Nascimento, Ana Grasiela Limoeiro, Carlos Eduardo Fontana

**Affiliations:** 1 Faculdade São Leopoldo Mandic, Instituto de Pesquisa São Leopoldo Mandic, Campinas, São Paulo, Brazil.; 2 Pontifícia Universidade de Campinas, Departamento de Endodontia, Escola de Ciências da Vida, Campinas, São Paulo, Brazil; 3 Faculdade de Odontologia de Bauru, Departamento de Dentística, Endodontia e Materiais Dentários, Universidade de São Paulo, Bauru, São Paulo, Brazil; 4 Pontifícia Universidade de Campinas, Programa de Pós-Graduação em Ciências da Saúde, Escola de Ciências da Vida, Campinas, São Paulo, Brazil

## Abstract

**Background:**

This study aimed to evaluate the cyclic fatigue resistance of WaveOne Gold (WOG) and R-Motion (RM) nickel-titanium instruments after multiple simulated clinical uses.

**Material and Methods:**

A total of 48 instruments (24 WOG and 24 RM) were tested. For each instrument system, files were divided into four subgroups (*n*=6 per subgroup) based on the number of prior simulated clinical uses: WOG0 and RM0 (new, no prior use), WOG1 and RM1 (single prior use), WOG2 and RM2 (two prior uses), and WOG3 and RM3 (three prior uses). Instrumentation was performed in 3D-printed resin teeths designed to simulate the mesial root canals of mandibular first molars. Subsequently, all instruments were subjected to a dynamic cyclic fatigue test in a stainless-steel artificial canal until fracture occurred. The number of cycles to fracture (NCF) was recorded. The groups had a normal distribution and were analyzed using the parametric ANOVA test, considering a 95% confidence interval, where the One-Way ANOVA, Bonferroni, Tukey, and Scheffe’ tests were used.

**Results:**

The time to fracture of the RM was higher when compared to the WOG (*p* < 0.05). There was no significant difference between the groups of the same instrument (*p* > 0.05). There was no significant difference between the length of the fractured instruments.

**Conclusions:**

Although both instruments showed good resistance to cyclic fatigue after 3 uses, the RM had better cycle fatigue resistance than the WOG.

** Key words:**Cyclic fatigue, Dental instruments, Endodontics, Root canal preparation.

## Introduction

Reciprocating systems used in endodontics were developed as a root preparation technique using a single file. Despite the advantages, there are still risks of fracture during root canal preparation, even if there is no visible deformation [1]. Stress and temperature influence the mechanical properties of endodontic instruments made from Nickel-Titanium (NiTi) alloy, which are sensitive to thermal cycles, modifying the microstructure [2]. In addition, clinical use, exposure to sodium hypochlorite (NaOCl) [3] and autoclave sterilization procedures [4,5] can affect their mechanical properties, reducing their resistance to cyclic fatigue.

According to the manufacturers, the instruments should be discarded after the first use. However, it seems reasonable to assume that the wear suffered by an instrument when used on a tooth with only 1 canal is less than that observed when used on a tooth with 3 canals [6]. Even with the risk of fracture, the reuse of reciprocating systems for more than one case is relatively common in clinical practice [7].

Cyclic fatigue occurs due to repeated compression and tension cycles of the instrument around the canal. To minimize these failures, manufacturers have developed strategies such as cross-sections, thermomechanical processes, and different kinematics [8].

The WaveOne Gold (WOG) reciprocating system (Dentsply Sirona, Ballaigues, Switzerland) has greater flexibility, resistance to cyclic fatigue and cutting efficiency. It has a parallelogram-shaped cross-section and a rounded tip. Its rotation is off-center, with only two points of contact between the instrument and the canal walls and, sequentially, only one point, reducing the risk of screwing [9]. The R-Motion (RM) instrument (FKG Dentaire, Switzerland) has a triangular cross-section with sharp edges, rounded free faces and an optimized tip promising to offer the perfect combination for greater cutting efficiency all the way to the apex, preserving the dentin. They are highly resistant to cyclic fatigue due to the heat treatment, which reduces the risk of instrument fracture, thus increasing safety for the professional and patient. The design and reduced size of the RM core significantly reduce the stress on the dentin during root canal treatment [10,11]. However, the efficiency of these instruments after reuse has not yet been scientifically substantiated.

The objective of this study was to determine the time to fracture of WaveOne Gold Primary 25.07 and R-Motion 25.06 instruments in simulated resin canals after 3 uses. The null hypotheses tested were that (i) there is no difference between the time to fracture of the instruments after reuse and (ii) there is no difference between the sizes of the broken instruments.

## Material and Methods

**Figure 1:**
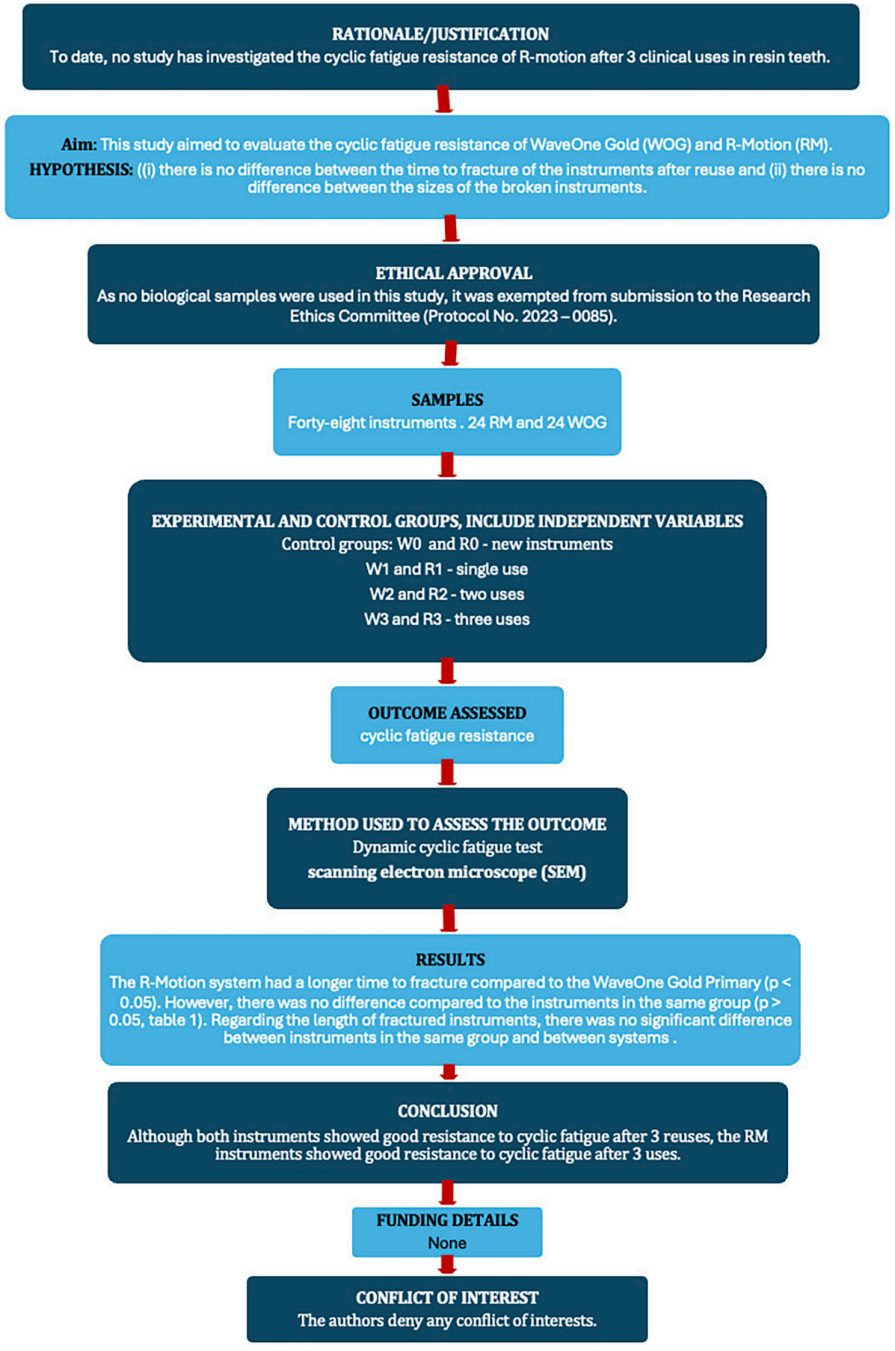
PRILE flowchart.

This study was submitted to and approved by the local ethics committee. The manuscript of this laboratory study was written according to the Preferred Reporting Items for Laboratory studies in Endodontology (PRILE) 2021 guidelines [12] (Fig. 1).

The sample for this study was calculated based on the effects observed in the study by Moreira *et al*. [7]. Based on this study, the effect size of the difference between groups was 2.19. Assuming a type I error of 5% (study confidence level of 95%), a type II error of 20% (study power of 80%), the effect size observed in the aforementioned study, and considering bilateral (two-sided) analyses, it was calculated that at least 6 sampling units were required in each of the experimental groups in the study.

- Root canal preparation

An endodontic specialist experienced in the use of the reciprocating instruments tested performed all laboratory procedures. Two NiTi reciprocating instruments were used: WaveOne Gold Primary (Dentsply Sirona) 25.07 with different and progressive taper characteristics and R-Motion (FKG) 25. 06 with reduced cores, both 25 mm long, driven by the x-Smart Plus electric motor (Dentsply Sirona) in WaveOne Gold mode for this file and in RECIPROC ALL mode for R-Motion, with three brush movements in and out for each third of the canal until the working length (WL) was reached. The WL was set at 1 mm below the apex. For the laboratory test, 72 artificial teeth were used, made of a resin suiTable for 3D printing and manufactured exclusively for this study. Tooth length was standardized at 22 mm, with a curvature of 57.4º in the mesial-vestibular, 24.73º in the mesial-lingual, and 29.5º in the distal region. A total of 24 instruments from each group were used, divided into four subgroups (*n*=06) as follows:

Group WOG (N=24):

WOG0 (Control Group): Brand new instruments were tested for cyclic fatigue without being used in any root canal procedures.

WOG1: New instruments were used for root canal preparation in six teeth, then tested for cyclic fatigue.

WOG2: New instruments were used for root canal preparation in six teeth. After this first use, the instruments were cleaned in an ultrasonic bath with enzymatic detergent for 180 seconds and sterilized in an autoclave at 134°C for 24 minutes. The rubber stop was removed with a sterile scalpel, and the instruments were then used in another six teeth (second use) before being tested for cyclic fatigue.

WOG3: The same protocol as WOG2 was followed, but after the second use (and another cleaning and sterilization cycle), the instruments were used for a third time in another six teeth before undergoing cyclic fatigue testing.

Group RM (N=24): The same procedures of the WOG groups.

- Subgroup division

A k#10 file was used 2 mm below the WL before reciprocal instrumentation and 1 mm beyond the apex between instrumentations for apical patency. 2.5% NaOCl was used for irrigation between instrumentations with a 29-G NaviTip needle (Ultradent, South Jordan, UT). The canals were kept filled with the irrigation solution, which was continuously renewed. A total of 25 mL of NaOCl was used for each tooth during each procedure. After laboratory preparation, the instruments were statistically evaluated for cyclic fatigue, fracture time, length of fractured instrument, visualization of the morphologic features of the instruments in the fracture area, and the type of fracture that occurred.

- Dynamic cyclic fatigue test

**Figure 2:**
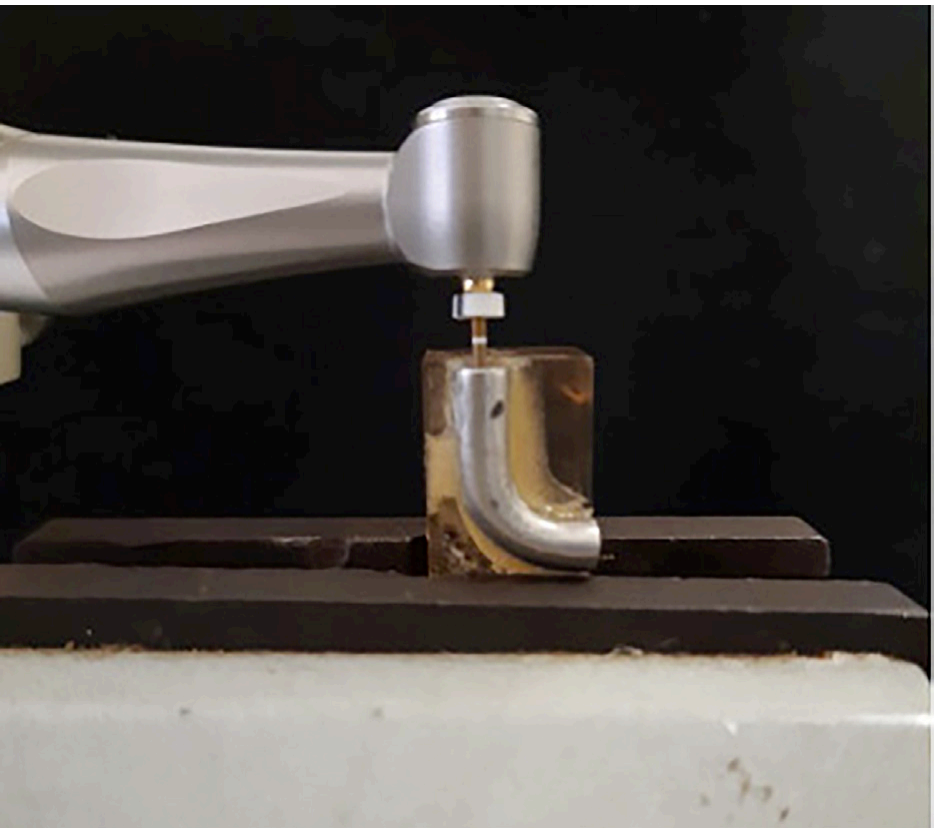
Device used in the fatigue tests of the instruments with a detail of the instrument inserted in the simulated canal.

To evaluate the mechanical behavior, the instruments were subjected to a cyclic fatigue test in a simulated root canal. The fatigue tests were performed using a device developed by the IME Biomaterials Laboratory team (Fig. 2). The device used simulated the clinical use of endodontic instruments by allowing vertical movements and simultaneous rotation of the instruments within the simulated canal.

**Figure 3:**
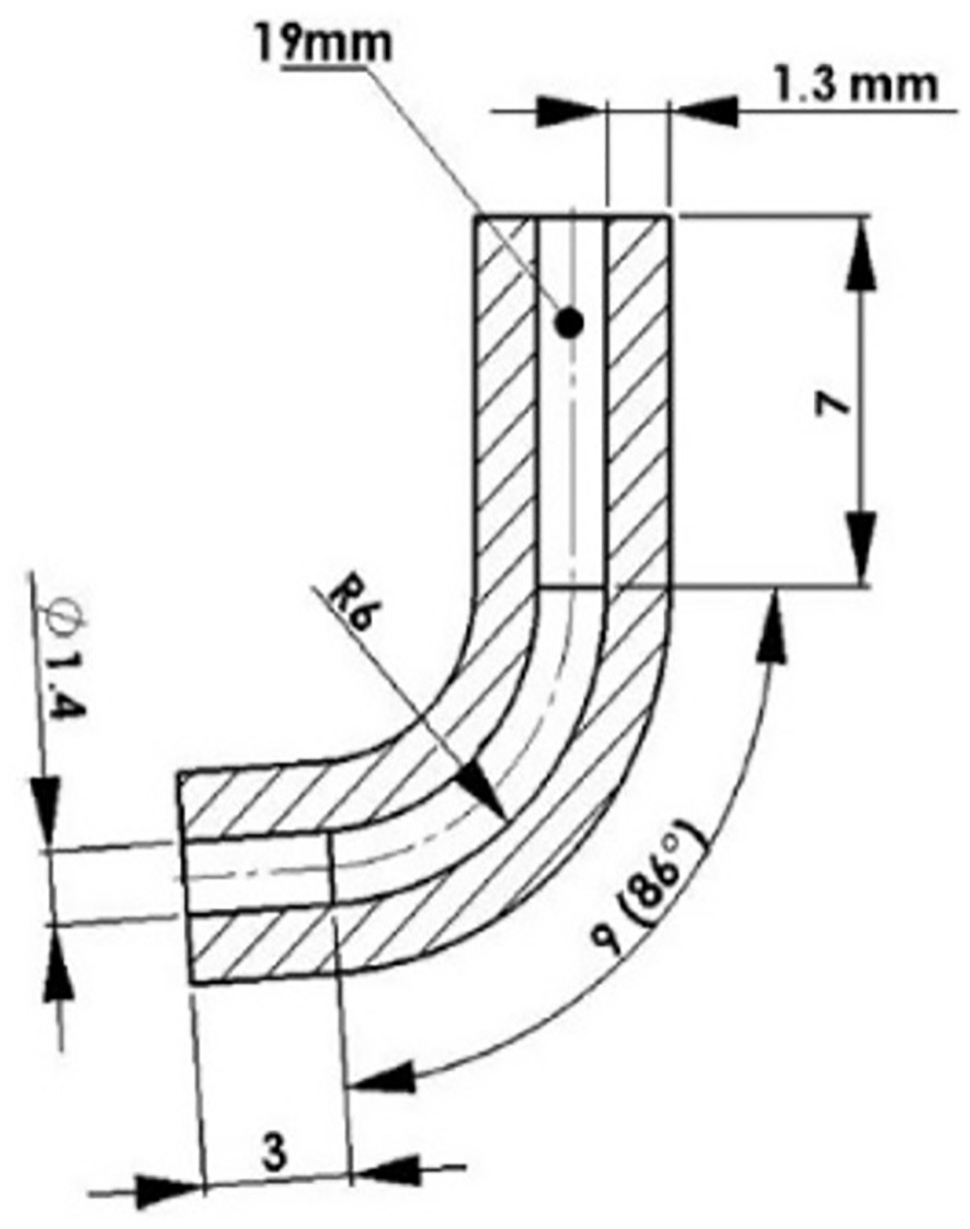
Detail of the canal used in the rotary bending test, showing its dimensions.

The simulated stainless-steel canal had an inner diameter of 1.4 mm, a total length of 19 mm, a curved segment with a radius of 6 mm, and a length of 9 mm in the curved section (Fig. 3). The instruments rotated freely in the canal, which was irrigated with 2.5% NaOCl and heated to 37°C. The test was interrupted if the instrument fractured inside the canal as soon as the fracture was detected visually and/or acoustically. The test time was calculated using a stopwatch. The number of cycles to fracture (NCF) was calculated by multiplying the rotational speed by the time (in seconds) to fracture. To calculate the NCF, one cycle of rotational loading was considered as one complete rotation of the instrument about its longitudinal axis. When the instrument is bent in the canal, at a rotation of 180o, the part that was in tension is compressed and vice versa. After a rotation of 360o, a bending cycle is completed, and the instrument returns to its initial position.

The tests were performed using a VDW Silver Reciproc motor (VDW GmbH, Munich, Germany) with a VDW 6:1 contra-angle handpiece (Sirona Dental Systems GmbH, Bensheim, Germany). For the fatigue tests, the instruments were subjected to different reciprocations according to the manufacturer’s instructions for use: “Wave One all” program for the WOG instruments and “Reciproc all” program for the RM instruments.

Analyses of the morphology of the instruments before and after the fatigue tests

**Figure 4:**
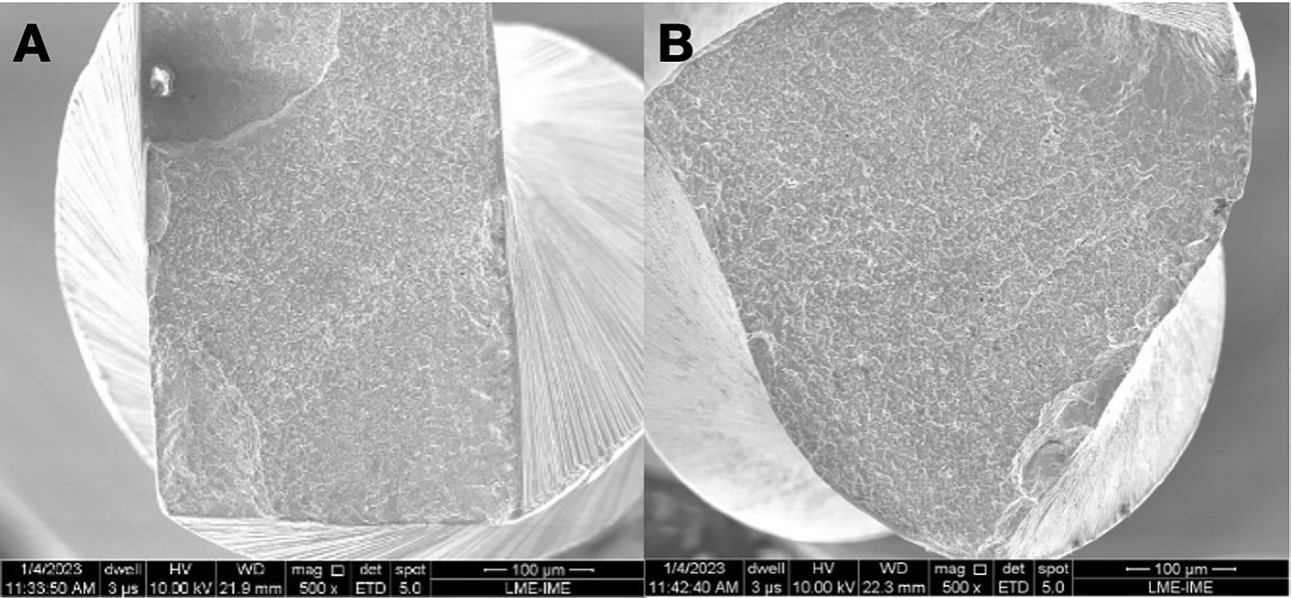
Fracture surface morphology of the WaveOne Gold Primary (A) and R-Motion (B) instruments. Morphology of 2 samples at magnification (500x).

Prior to the mechanical fatigue tests, two samples from each group of instruments were randomly selected for analysis of morphology and surface finish. The objective of the analysis was to identify the presence of possible manufacturing defects that could affect fatigue resistance. After the fatigue tests, the same samples were analyzed again (Fig. 4). The analysis was performed using a scanning electron microscope (FEI Quanta FEG250).

- Statistical analysis

Kolmogorov-Smirnov normality tests were performed. The groups were normally distributed and were analyzed using the parametric analysis of variance (ANOVA) test with a 95% confidence interval. Bonferroni, Student, and Tukey tests were used for the analysis. Considering the high standard deviation related to the mean fracture time, the test proposed by Weibull was used to determine the probability of instrument fracture as a function of duration of use.

## Results

The R-Motion system had a longer time to fracture compared to the WaveOne Gold Primary (*p* < 0.05). However, there was no difference compared to the instruments between groups of the same file brand (*p* > 0.05,  Table 1). Regarding the length of fractured instruments, there was no significant difference ( Table 2).

## Discussion

Although many endodontics instruments with different heat treatments are available on the market, there remain a risk of instrument fracture within the canal. For this reason, we tested two systems - R-Motion (RM) and Wave One Gold (WOG) – which, according to previous studies [11,13] exhibit great flexibility and resistance to cyclic fatigue. The instruments were reused up to three times and subjected to a dynamic cyclic fatigue test. There was a statistically significant difference between the fracture times of the RM and WOG instruments, so the first hypothesis was rejected. However, no significant difference was found in the fragment lengths of the broken instruments, so the second hypothesis was accepted.

Knowing the morphological and mechanical characteristics of endodontic instruments and using them correctly provides the user with more safety [14]. To minimize instrument fatigue, manufacturers have developed strategies such as different cross-sections design, kinematics, and introducing novel thermomechanical processes [15]. Some authors have shown that reciprocating motion provides higher cyclic fatigue resistance than rotary system [16,17]. Despite the indication for single use, several studies have shown that instruments can be safely reused [2,6,7,18]. This was also evident in the present study, as the files of both systems were reused up to three times without fracture.

RM instrument was compared with other instruments, and it showed greater capacity for canal centering, less canal transportation, and greater cutting efficiency [19]. Their resistance to fatigue is likely due to the reduced taper and metal mass due to their cross-section design, which also reduces dentin removal [10,20]. In our study, RM showed greater fatigue resistance than WOG. This result partially aligns with prior study [11], in which RM was more resistant to cyclic fatigue than OneCurve (Micro-Mega) but less resistant than Hyflex EDM (Coltene/Whaledent). Conversely, RM instrument was less resistant to dynamic cyclic fatigue than Race (FKG Dentaire SA, La Chaux de Fonds, Switzerland) [10]. Instruments with a larger cross-sectional area tend to have greater metal mass, impacting their resistance to fracture [20,21]; this may explain the findings with WOG. The fact that no files fractured in this study is likely due to the “gold” heat treatment, which was performed predominantly in the martensitic phase, and the design of the instrument, which reduces the risk of fatigue, a result that confirms previous studies [6,13]. Studies have shown that the fracture rate of reciprocating instruments is low when used by experienced endodontists [6,22].

All instruments showed a similar morphology, and the fracture surfaces showed typical characteristics of brittle fracture with the presence of microcavities, as in other studies [20,23].

Although static and dynamic cyclic fatigue tests on the same instrument can lead to conflicting results, a dynamic cyclic fatigue test was performed in this study, as suggested in previously published studies [10,15,18]. Another potential limiting factor for NiTi instrument strength is corrosion, which can occur during exposure to NaOCl and can negatively impact their physical and mechanical properties [24]. Interestingly, some studies have shown that cyclic fatigue resistance of NiTi instruments is not significantly affected by exposure to NaOCl or repeated sterilization cycles, repeated sterilization cycles [25,26], which contrasts with the results of another study in which cyclic fatigue was reduced after sterilization cycles [4]. For the rinse during the cyclic fatigue test, 2.5% NaOCl heated to 37 ºC was used, simulating the conditions normally used in clinical treatments. The temperature of the rinse may affect the flexural strength and cyclic fatigue of heat-treated instruments, as it has a higher martensite phase [27]. NiTi instruments rich in martensite can be easily deformed but recover their shape upon heating above the transformation temperature [28]. In addition, NiTi alloys in the martensitic state exhibit remarkable resistance to flexural fatigue [28,29].

Although a limitation of the study, it is worth noting that the use of artificial teeth allowed standardization of root canal anatomy, curvatures, and length of the tooth [30]. To reproduce the external morphology and root canal system, the teeth used were specially fabricated for this study and 3D printed to avoid distortion.

Further investigations employing extracted human teeth or conducted in clinical settings are necessary to determine if these results can be extrapolated to more complex root canal conFigurations and to assess the influence of varying operator experience. Although both instruments showed good resistance to cyclic fatigue after 3 reuses, the RM instruments showed good resistance to cyclic fatigue after 3 uses.

## Figures and Tables

**Table 1:** Time to instrument fracture (seconds).

	Without use (0)	One use (1)	Two uses (2)	Three uses (3)
WaveOne Gold	154.0 (± 39.2) Aa	155.8 (± 16.9) Aa	164.4 (± 20.0) Aa	158.4 (± 13.4) Aa
R-Motion	467.2 (± 65.9) Bb	446.8 (± 50.3) Bb	466.0 (± 64.9) Bb	400.8 (± 67.0) Bb

Caption: Capital letters indicate statistical difference horizontally; lower case letters indicate statistical difference vertically.

**Table 2:** Fractured fragment dimensions (mm).

	Without use (0)	One use (1)	Two uses (2)	Three uses (3)
WaveOne Gold	5.76 (± 0.11) Aa	4.46 (± 0.57) Aa	5.12 (± 0.16) Aa	5.00 (± 0.10) Aa
R-Motion	6.02 (± 1.53) Aa	5.56 (± 0.74) Aa	5.64 (± 1.63) Aa	5.66 (± 1.61) Aa

Caption: Capital letters indicate statistical difference horizontally; lower case letters indicate statistical difference vertically.

## Data Availability

The datasets used and/or analyzed during the current study are available from the corresponding author.
